# A novel mouse model of intrahepatic cholangiocarcinoma induced by liver-specific *Kras* activation and *Pten* deletion

**DOI:** 10.1038/srep23899

**Published:** 2016-04-01

**Authors:** Tsuneo Ikenoue, Yumi Terakado, Hayato Nakagawa, Yohko Hikiba, Tomoaki Fujii, Daisuke Matsubara, Rei Noguchi, Chi Zhu, Keisuke Yamamoto, Yotaro Kudo, Yoshinari Asaoka, Kiyoshi Yamaguchi, Hideaki Ijichi, Keisuke Tateishi, Noriyoshi Fukushima, Shin Maeda, Kazuhiko Koike, Yoichi Furukawa

**Affiliations:** 1 Division of Clinical Genome Research, Advanced Clinical Research Center, Institute of Medical Science, The University of Tokyo, Japan; 2 Department of Gastroenterology, Graduate School of Medicine, The University of Tokyo, Japan; 3 Division of Gastroenterology, Institute for Adult Diseases, Asahi Life Foundation, Japan; 4 Department of Cancer Genome Research, Sasaki Institute, Sasaki Foundation, Japan; 5 Department of Diagnostic Pathology, Jichi Medical University, Japan; 6 Department of Gastroenterology, Graduate School of Medicine, Yokohama City University, Japan.

## Abstract

Intrahepatic cholangiocarcinoma (ICC) is an aggressive malignancy with poor prognosis and its incidence is increasing worldwide. Recently, several types of cells have been considered as the origin of ICC, namely cholangiocytes, liver progenitor cells, and hepatocytes. Here, we have established a novel mouse model of ICC by liver-specific *Kras* activation and *Pten* deletion. An activating mutation of *Kras* in combination with deletion of *Pten* was introduced in embryonic hepatic bipotential progenitor cells (so-called hepatoblasts) and mature hepatocytes using the Cre-*loxP* system. As a result, liver-specific *Kras* activation and homozygous *Pten* deletion cooperated to induce ICCs exclusively. In contrast, *Kras* activation in combination with heterozygous *Pten* deletion induced both ICCs and HCCs, whereas *Kras* activation alone resulted in HCCs but not ICCs. Furthermore, a cell-lineage visualization system using tamoxifen-inducible Cre-*loxP* demonstrated that the ICCs did not originate from hepatocytes but from cholangiocytes. Our data suggest that mice carrying liver-specific *Kras* activation in combination with homozygous *Pten* deletion should be useful for the investigation of therapeutic strategies for human ICC.

Intrahepatic cholangiocarcinoma (ICC) is a primary epithelial neoplasm of the liver with characteristics of cholangiocyte differentiation. This highly malignant and progressive cancer ranks the second most common liver cancer in the world, and accounts for approximately 15% of all primary liver malignancies^1^. The incidence and mortality of ICC is reported to be increasing in several parts of the world, including North America, Europe, Australia, and Japan^2^,^3^,^4^.

Molecular studies have identified frequent mutations in *KRAS* in ICCs^5^,^6^,^7^,^8^. Ras signaling is deregulated in various human tumors, and approximately 30% of all human tumors have activating mutation in one of the *RAS* family genes. In particular, *KRAS* mutations are among the most common genetic abnormalities in human neoplasms, including pancreatic cancer^9^ and colon cancer^10^. The frequency of *KRAS* mutation shows a large variation among the tumor types. For example, the frequency has been reported to be less than 2% in hepatocellular carcinoma (HCC)^11^,^12^ and between 5 and 54% in ICC^6^,^7^,^8^,^13^. In addition, *BRAF*, an effector molecule of RAS, was reported to be mutated in a set of ICCs^14^. These studies provide strong evidence that activation of RAS-mediated signaling has an important role in the development of human ICC.

Activation of PI3K-AKT signaling is also involved in ICCs. The hyperphosphorylation of AKT, an indicator of the activation, has been frequently found in human ICC^15^. Furthermore, activating mutations in *PIK3CA* are observed in approximately 5% of ICCs^8^,^16^. This pathway recruits phosphatase and tensin homolog deleted on chromosome 10 (PTEN), a phosphoprotein/phospholipid dual-specificity phosphatase that preferentially dephosphorylates the second-messenger molecule phosphatidylinositol-3,4,5-trisphosphate, to antagonize the activity of PI3K. Loss of PTEN function also plays a vital role in a wide range of tumors through the constitutive activation of the PI3K-AKT pathway. Importantly, *PTEN* mutations were observed in ~11% of ICCs^8^,^17^, and inhibitory phosphorylation of PTEN has been reported in ICCs^18^. Hypermethylation of the CpG island in the *PTEN* promoter region was also reported in liver fluke-related ICCs^19^. In addition, microRNA-21 that inhibits PTEN is frequently overexpressed in ICCs^20^. These data suggest that inactivation of PTEN is one of the causes of activated PI3K/AKT signaling in human ICC.

Genetically engineered mouse models (GEMs) are essential tools to study the molecular pathogenesis of human diseases and assess novel therapeutic strategies preclinically. As a model of human pancreatic cancer, mice with pancreas-specific *Kras* activation were generated. The mice slowly developed premalignant pancreatic intraepithelial neoplasms (PanIN) with prolonged latencies and incomplete penetrance for the development of pancreatic ductal adenocarcinoma (PDAC)^21^. Furthermore, other models of rapidly progressing PDAC have been established by crossing the mice carrying pancreas-specific *Kras* activation with mice carrying loss of function in other genes that are associated with PDAC, such as *Ink4a/Arf*, *Trp53*, *Tgf* β *type II receptor*, or *Smad4*^22^,^23^,^24^,^25^.

In contrast to PDAC, a limited number of GEMs for ICC have been reported. These models include liver-specific *Pten* and *Smad4* double knockout, *Kras* activation in combination with *Trp53* knockout, inactivation of Hippo pathway by knockout of *Sav1*, and induction of intracellular domain of Notch receptor (NICD)^18^,^26^,^27^,^28^. It is noteworthy that most of these mouse models developed HCC as well as ICC because the genetic engineering was introduced in both hepatocytes and bipotential progenitor cells.

In the present study, we have established a novel genetically engineered mouse model of ICC. We found that *Kras* activation and homozygous *Pten* deletion in embryonic bipotential progenitor cells cooperate to exclusively induce ICC reminiscent of human ICC. Although several recent reports suggest the possibility that ICC can arise from hepatocytes through Notch-mediated transdifferentiation^29^,^30^, we have demonstrated, using cholangiocyte- or hepatocyte-specific Cre-*loxP* system, that ICCs in this model originated from cholangiocytes that were differentiated from *Kras* activated and *Pten* deleted progenitor cells.

## Results

### *Kras* activation and *Pten* deletion in hepatoblasts induce intrahepatic cholangiocarcinoma

**Figure 1:**
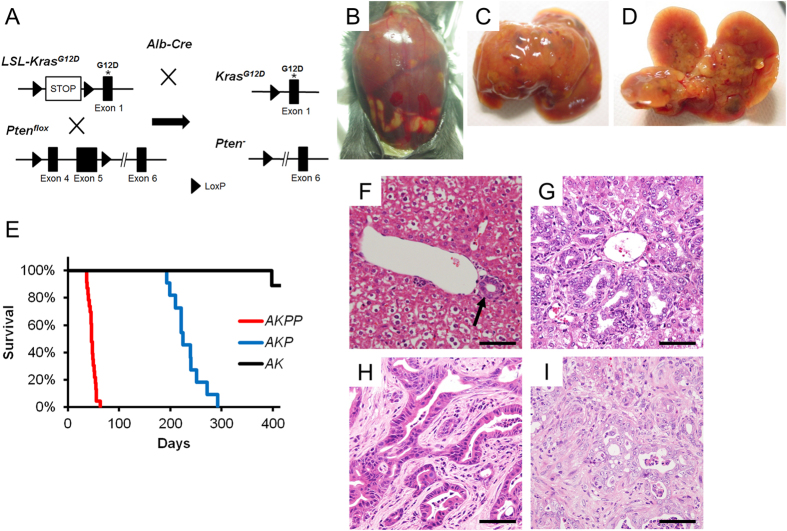
Generation of the mice with liver-specific *Kras*^*G12D*^ expression and *Pten* deletion. (**A**) Strategy to generate the compound mice. Conditional *Kras*^*G12D*^ knockin mice and conditional *Pten* knockout mice were crossed with *Alb-Cre* mice. (**B–D**) Gross appearance of an *AKPP* mouse at 8 weeks of age. Liver was enlarged and jaundice was observed, sometimes accompanying hemorrhagic ascites (**B**). Diffuse and firm tumorous lesions were observed in the liver (**C,D**). (**E–G**) H&E staining of the liver of the *AKPP* mice at 3 weeks, 5weeks, and 8 weeks of age. Normal bile duct formation (arrow) in the liver of a 3 week-old mouse (**E**). Bile duct hyperplasia in the liver of a 5 week-old mouse (**F**). Cholangiocarcinoma-like lesion in the liver of an 8 week-old mouse (**G**). Bars: 100 μm.

To investigate the role of cooperation of *Kras* activation and *Pten* inactivation in hepatotumorigenesis *in vivo*, we took advantage of conditional activation of a mutant *Kras* (*LSL-Kras*^*G12D*^) allele to express oncogenic Kras at a physiological level under endogenous *Kras* promoter after Cre-mediated recombination^31^, and conditional knockout allele of *Pten* (*Pten*^*flox*^)^32^. We crossed mice carrying a *LSL-Kras*^*G12D*^ allele and/or a *Pten*^*flox*^ allele with mice expressing Cre recombinase under the control of albumin promoter (*Alb-Cre*)^33^ to express the oncogenic *Kras*^*G12D*^ allele and/or to delete *Pten* alleles specifically in the liver (Fig. 1A).

We first analyzed macroscopic phenotype of liver and survival in *Alb-Cre*^+^; *LSL-Kras*^*G12D/*+^; *Pten*^*flox/flox*^ mice (*AKPP* mice), *Alb-Cre*^+^; *LSL-Kras*^*G12D/*+^; *Pten*^*flox/*+^ mice (*AKP* mice), and *Alb-Cre*^+^; *LSL-Kras*^*G12D/*+^; *Pten*^+*/*+^ mice (*AK* mice). In these mice, both *Pten* alleles (*AKPP* mice), one *Pten* allele (*AKP* mice), and no *Pten* allele (*AK* mice) was deleted in combination with *Kras* activation specifically in the liver. Mice with these genotypes were obtained at the expected Mendelian frequency at birth but the *AKPP* mice alone showed apparent growth retardation. All of the *AKPP* mice started to demonstrate abdominal distension at ~5 weeks of age, frequently accompanied by jaundice and weight loss. The distension was caused by hepatic enlargement and/or hemorrhagic ascites. These manifestations recapitulated well those frequently observed in human ICC. They were euthanized when they appeared to be in distress. Autopsies revealed the presence of multiple solid tumors with various sizes throughout the liver (Fig. 1B–D). The median survival was 46 days (Fig. 1E).

*AKP* and *AK* mice also developed liver tumors at 6 and 12 months, respectively (Fig. 1E). Although the majority of *AKP* mice did not show obvious symptoms until 7 months of age, they suffered from abdominal distension due to multiple liver tumors at later stages. On the other hand, *AK* mice did not show obvious symptoms up to an age of 18 months (Fig. 1E).

We next evaluated histological changes of the liver tumors in *AKPP* mice. Although no apparent abnormality was histologically found in the liver of *AKPP* mice until 3 weeks of age (Fig. 1F), various degrees of hyperplasia in the bile ducts, characterized by increase in number and size, and change in morphology, were observed at 4 weeks of age. At 5 weeks, a part of the hyperplastic ductal lesions at the hilum became enlarged and showed the pattern of papillary growth, and the number of dysplastic ducts was increased (Fig. 1G). After 7 weeks, invasive tumors were apparent with an abundant desmoplastic stroma, or “desmoplasia” (Fig. 1H). The tumors primarily showed glandular morphology that resembled well-differentiated human CC. In addition, regions of moderately differentiated tumor with a cribriform appearance were observed in some cases (Fig. 1H,I). No apparent metastasis or invasion to other organs was observed in *AKPP* mice.

**Figure 2:**
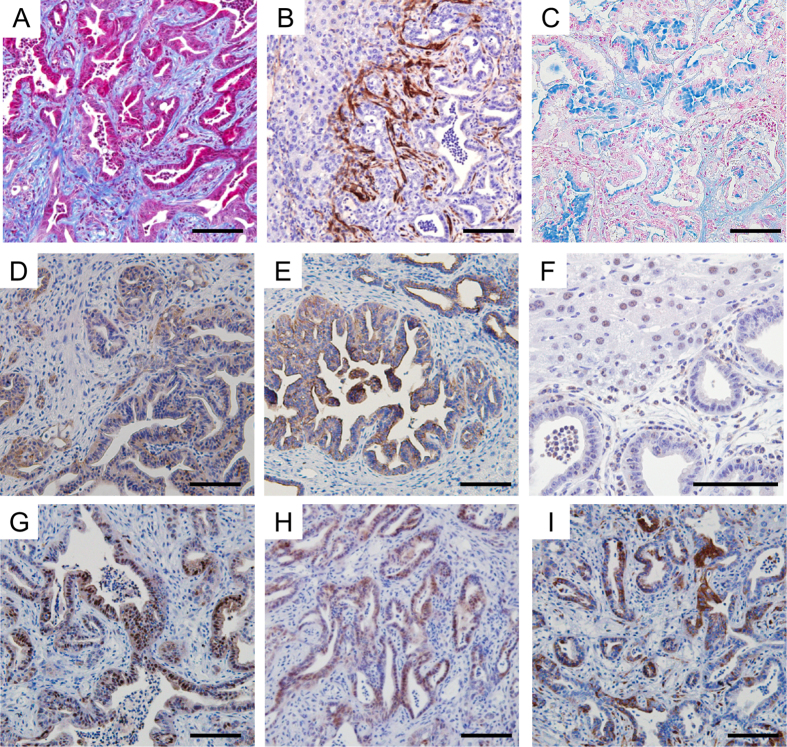
Characterization of the liver tumors in the *AKPP* mice. (**A–C**) The expression of cholangiocyte and hepatocyte markers. (**A,B**) Masson-trichrome staining (**A**) and αSMA immunohistochemical staining (**B**) showed fibrosis in the tumor tissues. (**C**) Alcian blue staining showed that some of the tumor cells produced mucin. (**D–F**) Cholangiocyte markers, pan-Ck (**D**) and Ck19 (**E**) were positive, while a hepatocyte marker, Hnf4α (**F**) was negative in the tumor cells by immunohistochemical staining. (**G**) Immunohistochemical staining of p-Erk showed activation of Mapk pathway in the tumor cells. (**H**,**I**) Immunohistochemical staining of p-Akt (**H**) and p-S6 (**I**) showed activation of Pi3k-Akt-mTorc1 pathway in the tumor cells. Bar: 100 μm.

The presence of desmoplasia around the tumor is generally considered to be one of the most characteristic histological findings for distinguishing ICC from HCC^34^. The Masson-Trichrome staining frequently depicted tumors surrounded by dense fibrous stroma in *AKPP* mice, indicating the accumulation of fibrillar collagens (Fig. 2A). Immunohistochemical analysis with an anti-αSMA antibody showed positive staining in the stroma surrounding the tumors, which implied that the stellate cells were activated and acquired a myofibroblast-like phenotype (Fig. 2B). Alcian blue staining exhibited mucin production in the tumor cells (Fig. 2C), suggesting that they have characteristics of epithelial cells in the bile duct.

To further clarify the characteristics of the tumor cells developed in *AKPP* mice, we analyzed various markers used for diagnosis of ICC. The tumor cells showed positive staining for pan-cytokeratin (pan-Ck) and Ck19 (Fig. 2D,E)^35^, markers of cholangiocyte differentiation. On the other hand, they were negative for Hnf4α, a specific marker of hepatocyte differentiation (Fig. 2F)^35^. These results demonstrated that *Kras* activation and *Pten* homozygous deletion cooperate to induce ICC exclusively.

To confirm the activation of Mapk and Pi3k pathways in the tumors of the *AKPP* mice, immunohistochemical staining of p-Erk and p-Akt was conducted. As expected, the tumor cells were positive for both p-Erk and p-Akt (Fig. 2G,H). In addition, immunohistochemical staining of p-S6, a marker for the activation of mTorc1, was positive in the tumor cells (Fig. 2I). These results suggested that Mapk and Pi3k-mTorc1 pathways were activated in the ICC induced by *Kras* activation and *Pten* deletion.

In contrast to the tumors in *AKPP* mice, *AKP* mice developed liver tumors with both hepatocyte and cholangiocyte differentiation. Histological examination revealed that the majority of large nodules in the *AKP* mice were hepatocellular dysplasia (HD), which is considered as a precursor lesion of HCC (Figure S1A). Partially, there were lesions diagnosed as ICC by the appearance of adenocarcinoma showing tubular and/or papillary structures with a fibrous stroma (Figure S1B). Liver parenchyma surrounding these tumors was not cirrhotic. Interestingly, immunohistochemical staining of p-Erk and p-Akt revealed the activation of Mapk pathway, but not the activation of Pi3k pathway, in HDs (Figure S2A,C), whereas coactivation of these two pathways was detected in ICC lesions (Figure S2B,D). In addition, loss of Pten expression was shown in the tumor cells of ICCs in *AKP* mice by immunohistochemical staining (Figure S3A,B), suggesting that the remaining wild-type *Pten* allele was inactivated by some mechanism such as loss of heterozygosity, loss-of-function mutation, and hypermethylation of its promoter region.

Regarding *AK* mice, they showed no abnormality until 12 months of age, but they later exhibited abdominal distension due to the enlarged liver with multiple tumors. Histopathological examination revealed that the tumors resembled HD that developed in the *AKP* mice (Figure S1C). No abnormality in biliary system was detected in the liver of the *AK* mice.

Collectively, in combination with oncogenic Kras expression, Pten dose might dictate the fate determination of hepatotumorigenesis into biliary or hepatocyte cell lineage. In addition, it is noteworthy that homozygous *Pten* deletion in cooperation with oncogenic *Kras* mutation induces ICC exclusively.

### *Kras* activation and *Pten* deletion in cholangiocytes but not hepatocytes induce ICC

**Figure 3:**
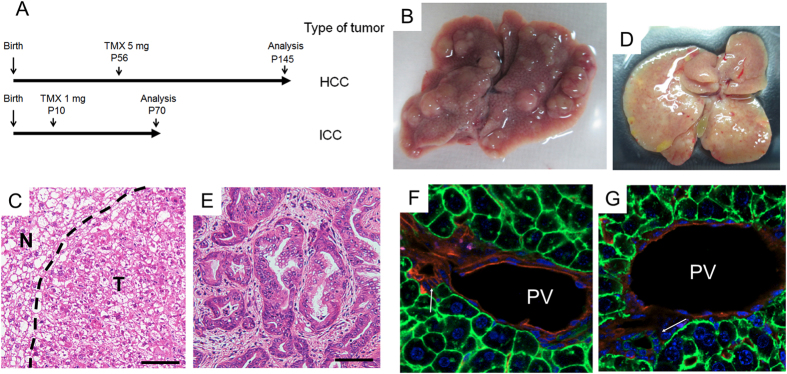
Analysis of cell origin of the ICC induced by liver-specific *Kras* activation and *Pten* deletion. (**A**) Experimental design for induction of liver tumors in *A*^*ER*^*KPP* mice with two different times of tamoxifen (TMX) treatment. (**B**) Appearance of the liver in an *A*^*ER*^*KPP* mouse treated with TMX at P56 and then euthanized 3 months later showed multiple solid tumors on the liver surface. (**C**) Representative histological appearance of liver tumors in (**B**) showed they were HCCs and HDs. (**D**) Appearance of the liver in an *A*^*ER*^*KPP* mouse treated with TMX at P10 and then euthanized 2 months later showed diffuse and hard tumors. (**E**) Representative histological appearance of liver tumors in (**D**) showed they were ICCs. (**F,G**) Analysis of TMX-induced recombination of liver cells in *Alb-CreERT2*; *mTmG* mice. The *Alb-CreERT2*; *mTmG* mice were treated with TMX at P56 (**F**) or P10 (**G**) and then analyzed one week later. None of the cells that formed bile ducts were positive for EGFP (green) with TMX at P56, indicating that Cre-*loxP* mediated gene recombination occurred only in hepatocytes (**F**). In contrast, the majority of the bile duct cells were positive for EGFP (green) with TMX at P10, indicating that gene recombination occurred not only in hepatocytes but also in cholangiocytes (**G**). Red: tdTomato, Blue: DAPI. Bars: 100 μm.

To elucidate whether mature hepatocytes can be the origin of ICC induced by *Kras* activation and *Pten* deletion, we crossed *LSL-Kras*^*G12D/*+^; *Pten*^*flox/flox*^ mice with *Alb-CreERT2*^+^ mice in which tamoxifen (TMX)-inducible *CreERT2* was knocked into endogenous *Alb* locus^36^. These *Alb-CreERT2*^+^; *LSL-Kras*^*G12D/*+^; *Pten*^*flox/flox*^ mice (*A*^*ER*^*KPP* mice) were injected with TMX at eight weeks after birth and tumor development was investigated three months later (Fig. 3A). Consequently, multiple liver tumors were observed in these mice (Fig. 3B). Histological analyses showed that all tumors were HCC and HD but not ICC (Fig. 3C). These results suggest that *Kras* activation and *Pten* deletion in differentiated hepatocytes induce HCC but not ICC.

We next administered TMX to *A*^*ER*^*KPP* mice at postnatal day 10 (P10) to investigate the possibility that hepatocytes in young mice could be the origin of ICC. Interestingly, these mice developed ICCs exclusively, which phenocopied the *AKPP* mice (Fig. 3D,E).

The fact that the different timing of TMX administration altered the types of liver tumor prompted us to examine the possibility of the age-dependent difference of cell types where Cre-mediated recombination occurred in the *A*^*ER*^*KPP* mice. To test this possibility, we generated *Alb-CreERT2*^+^; *Rosa26-mTmG* reporter (*Alb-CreERT2*^+^; *R26R*^*mTmG/*+^) mice by crossing *Alb-CreERT2* mice with *R26R*^*mTmG*^ mice that express membrane-targeted EGFP (mG) in Cre-expressing cells and membrane-targeted tdTomato (mT) in Cre-absent cells^37^. Examination of the liver in *Alb-CreERT2*^+^; *R26R*^*mTmG/*+^ mice with TMX-administration at eight weeks after birth depicted EGFP expression only in the hepatocytes (Fig. 3F). In contrast, in the mice administered with TMX at P10, EGFP expression was detected not only in the hepatocytes but also in the cells that form the bile ducts (Fig. 3G). These results suggest that ICCs originated from the cholangiocytes carrying Cre-mediated *Kras* activation and *Pten* deletion in the *A*^*ER*^*KPP* mice.

### *Kras* activation and *Pten* deletion in mature choalngiocytes induce ICC

**Figure 4:**
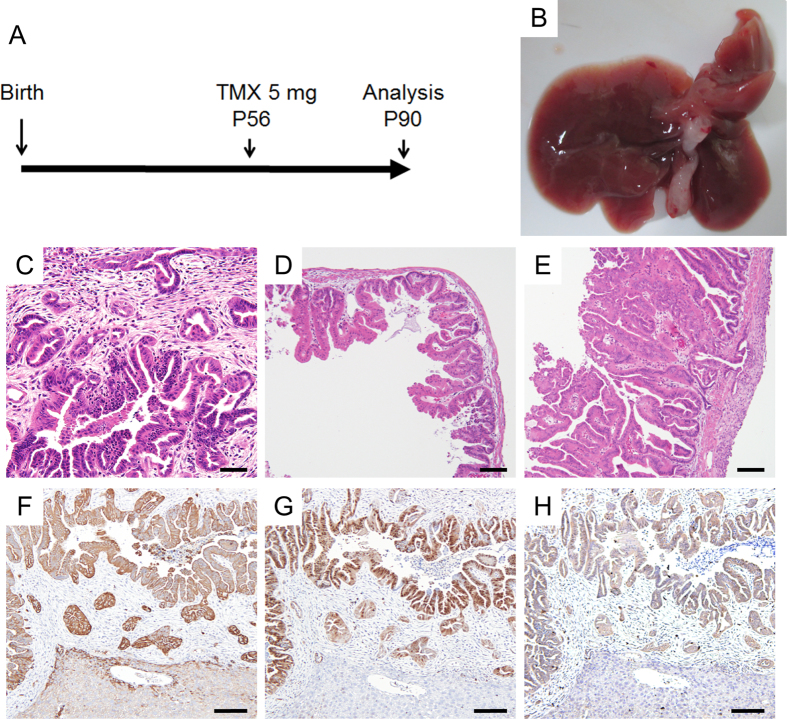
ICC induction by cholangiocyte-specific *Kras* activation and *Pten* deletion. (**A**) Experimental design induction of liver tumors in *K*^*ER*^*KPP* mice with tamoxifen (TMX) treatment. (**B**) Gross appearance of the liver of a *K*^*ER*^*KPP* mouse 5 weeks after TMX treatment. Note that no obvious liver tumor was observed but the GB and the extrahepatic bile duct (EBD) were severely dilated. (**C–E**) Histologically, ICC-like lesions were found in the liver (**C**). Papillary hyperplastic lesions were seen in GB (**D**) and EBD (**E**). (**F**) The ICC-like lesions were positive for immunohistochemical staining of pan-Ck. (**G,H**) Immunohistochemical staining of p-Erk (**G**) and p-Akt (**H**) revealed activation of Mapk and Pi3k pathways in the lesions. Bars: 100 μm.

To investigate whether *Kras* activation and *Pten* deletion in intrahepatic bile ducts induce ICC, we took advantage of mice with a TMX-inducible Cre under the control of *Ck19* promoter (*K19*^*CreERT*^)^38^. We crossed them with *LSL-Kras*^*G12D*^ mice and *Pten*^*flox*^ mice to generate *K19*^*CreERT/*+^; *LSL-Kras*^*G12D/*+^; *Pten*^*flox/flox*^ mice (*K*^*ER*^*KPP* mice). We administered TMX at eight weeks of age (Fig. 4A). The median survival of the *K*^*ER*^*KPP* mice was 30 days after TMX injection. Although no obvious tumors were macroscopically observed in the liver, their extrahepatic bile duct as well as the gallbladder and cystic duct were rigid and dilated (Fig. 4B). Histological analysis of the liver revealed pre-malignant papillary ductal lesions in periportal areas (Fig. 4C). Besides the ductal lesions in their liver, a variety of organs showed pathological abnormalities, including papillary hyperplasia of the extrahepatic biliary tract and gallbladder, PanIN-like lesions and hyperplasia in the pancreatic ducts, hyperplastic changes of gastric mucosa, colonic serrated epithelial changes, and papillary hyperplasia of bronchial epithelia that accompanied obstructive pneumonia (Fig. 4D,E and Figure S4A,B). Respiratory failure by the lung lesions was most likely to be the cause of death of the *K*^*ER*^*KPP* mice. The hepatobiliary phenotypes in the *K*^*ER*^*KPP* mice were consistent with those reported in *Ah-CreER*^*T/*+^; *LSL-Kras*^*G12V/*+^; *Pten*^*flox/flox*^ mice, in which β-naphthoflavone/TMX-inducible *Kras* activation and *Pten* deletion were introduced in biliary as well as gastrointestinal epithelia^39^. The premalignant ductal lesions in the liver were positive for pan-Ck (Fig. 4F), confirming that *Kras* activation and *Pten* deletion is capable of inducing ICCs from cholangiocytes. In addition, we observed positive immunohistochemical staining with both P-Erk and P-Akt antibodies in the neoplastic cells (Fig. 4G,H), suggesting that Mapk and Pi3k pathways were activated by the activation of *Kras* and the deletion of *Pten*.

## Discussion

In this study, we have established a novel mouse model of ICC by liver-specific *Kras* activation and *Pten* deletion (*AKPP*). The *AKPP* mice, in which *Kras* activation and homozygous deletion of *Pten* were introduced specifically in the liver, developed exclusively ICCs. There have been several reports of genetically engineered mouse models of ICC but most of the models develop HCC as well as ICC^18^,^26^,^27^. Similarly, the *AKP* mice, in which *Pten* allele was heterozygously inactivated, developed both HCC and ICC. It has been reported that liver-specific homozygous deletion of *Pten* induces HCC as well as ICC in mice at more than 12 months of age^18^,^40^. These reports suggest two possible explanations for our observation that the *AKPP* mice exclusively developed ICCs. One is that the limited life-span of the *AKPP* mice (median survival: 46 days) was not long enough to develop HCCs. The other is that excessive activation of Pi3k pathway in combination with oncogenic Kras expression promotes the formation of ICC, but not the formation of HCC. In agreement with the latter possibility, immunohistochemical analysis revealed that the activation of Pi3k pathway was observed in the ICCs but not in HCCs/HDs in the *AKP* mice. Since Pten expression was lost in the tumor cells of ICCs in the *AKP* mice, ICCs were induced in the mice through the biallelic inactivation of *Pten* in combination with *Kras* activation as observed in the *AKPP* mice. It is of note that the *AK* mice with oncogenic *Kras* along with intact *Pten* expression in the liver developed HCCs/HDs but not ICCs. Therefore, the activation of Pi3k pathway might determine the fate of tumorous cells caused by oncogenic Kras from hepatocyte linage to biliary lineage.

Investigating the cell origin of ICC has been considered to be important for the development of new therapies for this devastating disease. ICC has been recently classified into three groups, pure mucin-producing ICC (muc-ICC), predominant mucin-producing ICC with hepatocytic differentiation areas and/or ductular areas in the tumor (mixed-ICC), and predominant ductular areas with hepatocytic differentiation areas with muc-producing ICC areas (cholangiolocellular carcinoma; CLC)^41^. Muc-ICCs are thought to be of cholangiocyte origin, whereas mixed-ICC and CLCs are thought to originate from hepatic progenitor cells^41^, by their clinicopathological and molecular features. Meanwhile, several groups have reported that ICCs can originate from differentiated hepatocytes by transdifferentiation^29^,^30^. In the present study, we have shown that the ICCs were induced by liver-specific *Kras*^*G12D*^ expression and *Pten* deletion using *Alb-Cre* mice, *Alb-CreERT2* mice with TMX-administration at P10, and *K19*^*CreERT*^ mice with TMX-administration at P42. In contrast, no ICCs were induced using *Alb-CreERT2* mice with TMX-administration at P42. TMX-administration at P10 was shown to cause Cre-*loxP*-mediated gene recombination in bile duct cells as well as hepatocytes, whereas TMX-administration at P42 caused the recombination only in hepatocytes. Taken together, these results suggest that cholangiocytes are likely to be the origin of ICC in these models. In agreement with our results, Guest *et al.* have recently revealed that biliary epithelia is a possible origin of ICC using cholangiocyte-lineage tracing system to induce p53 loss in biliary epithelia in combination with chronic liver injury^42^. Although the ICCs in the *AKPP* mice were originated from cholangiocytes like muc-ICCs in human, they do not so frequently produce mucin as muc-ICCs. This finding suggests that *Kras* activation and *Pten* deletion are not enough to the induction of mucin production in biliary epithelia. Alternatively, murine biliary epithelia might be insensitive to intracellular signaling that promote mucin production. Nevertheless, the *AKPP* mice should be a useful model for the investigation of molecular mechanisms of the development of ICC from cholangiocytes.

Comprehensive genome profiling of intrahepatic cholangiocarcinoma has been recently reported by several groups^8^,^12^,^43^,^44^,^45^,^46^. These studies revealed that *TP53* and *KRAS* are two of the genes most frequently mutated in human ICC. In addition, Zou *et al.* revealed that the RAS/PI3K pathway-related genes were frequently altered by the pathway analysis of 103 ICC patients^8^. Consistent with these data, an inactivating mutation of *PTEN* or an activating mutation of *PIK3CA*, the two representative mutations that activate the PI3K pathway, was shown to coexist with oncogenic *KRAS* mutation in a subset of human ICCs^8^. The *AKPP* mice should be the most suitable mouse model to facilitate the development of new therapies for ICCs with an oncogenic *KRAS* mutation and the activated PI3K pathway.

In conclusion, we have established a novel mouse model of ICC by introducing the oncogenic *Kras* mutation and the homozygous *Pten* deletion in the liver. The tumors induced in this model were exclusively ICCs that histologically recapitulate human ICC of cholangiocyte origin. Intensive investigation of this model should provide better understanding of human ICCs derived from biliary epithelial cells and facilitate the development of new therapies for this devastating disease.

## Materials and Methods

### Mice

*Alb-Cre*, *LSL-Kras*^*G12D*^, and *R26R*^*mTmG*^ mice were purchased from The Jackson Laboratory^31^,^33^,^37^. *Pten*^*flox*^, *Alb-CreERT2*, and *K19*^*CreERT*^ mice were kindly provided by Tak W Mak (University of Toronto, Toronto, Canada), Daniel Metzger and Pierre Chambon (IGBMC, Illkirch-Cedex, France), and Guoqiang Gu (Vanderbilt University, Nashville, TN), respectively^32^,^36^,^38^. All mice were on the C57BL/6 genetic background. PCR primers used for the genotyping of each mouse strain are listed in Supplemental Table S1. All mice were housed in specific pathogen-free conditions within the animal care facility at the University of Tokyo. All the experimental protocols were approved by the Ethics Committee for Animal Experimentation and conducted in accordance with the Guidelines for the Care and Use of Laboratory Animals of the Institute of Medical Science and Department of Medicine, the University of Tokyo.

### Tamoxifen treatment of mice

For the induction of tumors in the *A*^*ER*^*KPP* mice, the mice were treated with 1 mg and 5 mg of tamoxifen (TMX) at P10 and P56, and then euthanized 2months and 3 months later, respectively. For tumor induction in the *K*^*ER*^*KPP* mice, the mice were treated with 5 mg of TMX at P56 and then euthanized one month later.

To analyze the origin of tumors in the *A*^*ER*^*KPP* mice, the *Alb-CreERT2* mice were treated with 1 mg and 5 mg of TMX at P10 and P56 and then euthanized one week later.

### Histology and immunohistochemistry

Normal and tumor tissues were fixed in 4% paraformaldehyde overnight then processed, paraffin-embedded, sectioned and stained with hematoxylin and eosin, Masson-trichrome, and Alcian blue according to standard protocols. For immunohistochemistry, 5 μm sections were incubated with primary antibodies overnight at 4 °C in a humidified chamber. The following primary antibodies were used: HNF4α (Santa Cruz), CK19 (TROMAIII, Developmental Hybrydoma Bank), Pan-cytokeratin (AE1/AE3, GeneTex), p-AKT^Ser473^ (Cell Signaling), p-ERK (Cell Signaling), p-S6 (Cell Signaling), PTEN (Cell Signaling), and αSMA (Sigma). For rabbit and rat antibodies, the sections were subsequently developed using a VECTASTAIN Elite ABC kit (Vector Laboratories). For mouse monoclonal staining, a MOM kit (Vector Laboratories) was also used. Representative sections from at least three mice were counted for each genotype. For immunofluorescence, liver tissues with mTmG expression were fresh-frozen in Optimal Cutting Temperature medium (Tissue-Tek). 5 μm sections were fixed with methanol for 10 min at −20 °C, washed 3 times with PBS, mounted in VECTASHIELD mounting medium with DAPI (Vector Laboratories), and imaged with confocal microscopy (Nikon).

## Additional Information

**How to cite this article**: Ikenoue, T. *et al.* A novel mouse model of intrahepatic cholangiocarcinoma induced by liver-specific *Kras* activation and *Pten* deletion. *Sci. Rep.* **6**, 23899; doi: 10.1038/srep23899 (2016).

## Supplementary Material

Supplementary Information
