# Corrigendum: A novel mouse model of intrahepatic cholangiocarcinoma induced by liver-specific *Kras* activation and *Pten* deletion

**DOI:** 10.1038/srep39567

**Published:** 2017-01-03

**Authors:** Tsuneo Ikenoue, Yumi Terakado, Hayato Nakagawa, Yohko Hikiba, Tomoaki Fujii, Daisuke Matsubara, Rei Noguchi, Chi Zhu, Keisuke Yamamoto, Yotaro Kudo, Yoshinari Asaoka, Kiyoshi Yamaguchi, Hideaki Ijichi, Keisuke Tateishi, Noriyoshi Fukushima, Shin Maeda, Kazuhiko Koike, Yoichi Furukawa

Scientific Reports 6: Article number: 2389910.1038/srep23899; published online: 04 01 2016; updated: 01 03 2017

This Article contains an error in the Figure legend of Figure 1. The correct Figure legend is given below:

**Figure 1. Generation of the mice with liver-specific KrasG12D expression and Pten deletion.** (**A**) Strategy to generate the compound mice. Conditional KrasG12D knockin mice and conditional Pten knockout mice were crossed with Alb-Cre mice. (**B–D**) Gross appearance of an AKPP mouse at 8 weeks of age. Liver was enlarged and jaundice was observed, sometimes accompanying hemorrhagic ascites (**B**). Diffuse and firm tumorous lesions were observed in the liver (**C,D**). (**E**) Survival of AKPP (n = 23), AKP (n = 11), and AK (n = 9) mice. (**F–I**) H&E staining of the liver of the AKPP mice at 3 weeks, 5weeks, and 8 weeks of age. Normal bile duct formation (arrow) in the liver of a 3 week-old mouse (**F**). Bile duct hyperplasia in the liver of a 5 week-old mouse (**G**). A highly differentiated cholangiocarcinoma-like lesion in the liver of an 8 week-old mouse (**H**). A moderately differentiated cholangiocarcinoma-like lesion in in the liver of an 8 week-old mouse (**I**). Bars: 100μm.

