# Supplementary material for: A novel mouse model of intrahepatic cholangiocarcinoma induced by liver-specific Kras activation and Pten deletion
Source: Sci Rep. 2016 Apr 1;6:23899. doi: 10.1038/srep23899 (PMC4817147; doi:10.1038/srep23899)
Supplement: Supplementary Information [file srep23899-s1.doc]

**A novel mouse model of intrahepatic cholangiocarcinoma by liver-specific *Kras* activation and *Pten* deletion**

Tsuneo Ikenoue, Yumi Terakado, Hayato Nakagawa, Yohko Hikiba, Tomoaki Fujii, Daisuke Matsubara, Rei Noguchi, Chi Zhu, Keisuke Yamamoto, Yotaro Kudo, Yoshinari Asaoka, Kiyoshi Yamaguchi, Hideaki Ijichi, Keisuke Tateishi, Noriyoshi Fukushima, Shin Maeda, Kazuhiko Koike, Yoichi Furukawa

**
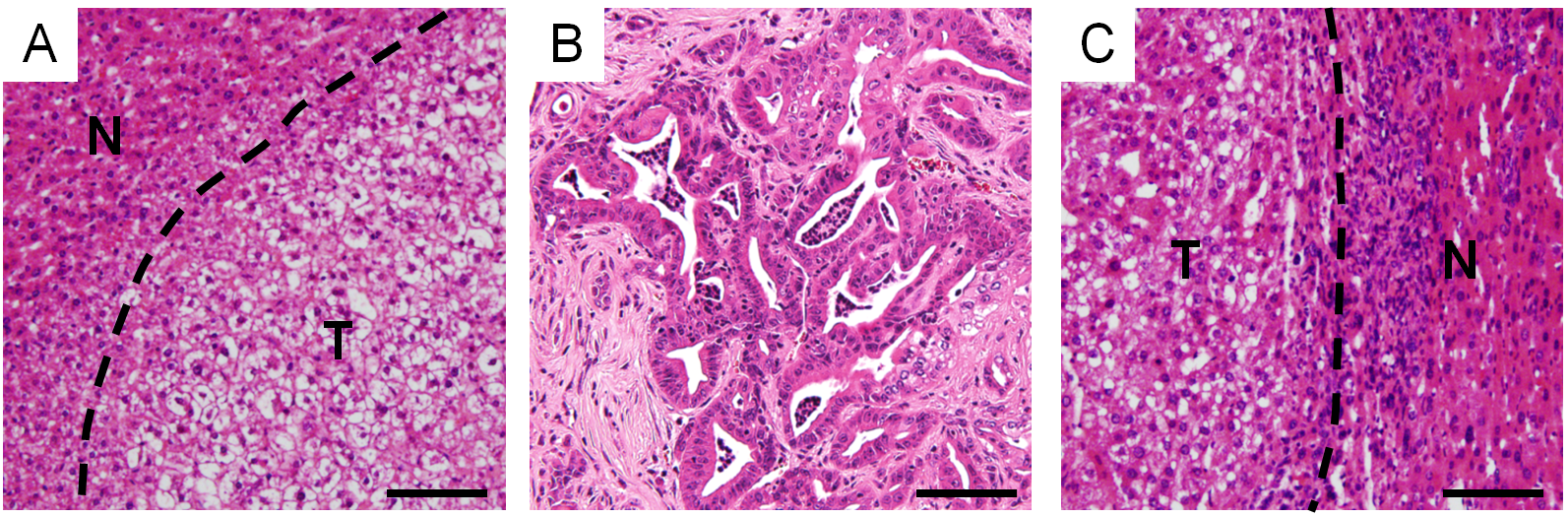
**

**Supplementary Figure S1.** Histology of liver tumors by liver-specific *Kras* activation with heterozygous *Pten* deletion or liver-specific *Kras* activation alone. (A) A hepatocytic dysplastic nodule in an *AKP* mouse at 8 months of age. (B) A tumor with cholangiocarcinoma-like appearance in an *AKP* mouse at 7 months of age. (C) A hepatocytic dysplastic nodule in an *AK* mouse at 12 months of age. Bar: 100 μm.


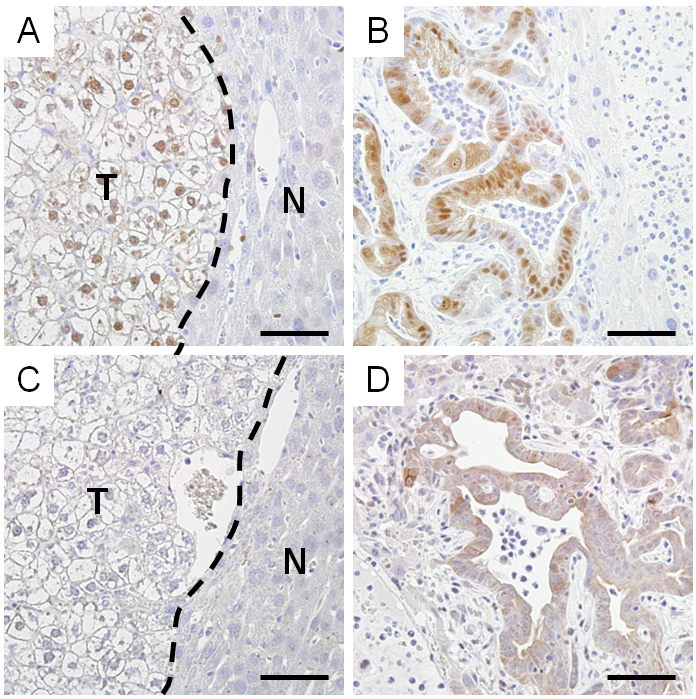


**Supplementary Figure S2.** Representative pictures of immunohistochemical staining of p-Erk (A, B) and p-Akt (C, D) in dysplastic nodules (A, C) and ICC-like lesions (B, D) in *AKP* mice at 7-8 months of age. In the dysplastic nodules, p-Erk was positive (A), but p-Akt was negative (C) in the tumorous cells. In the ICC-like lesions, both p-Erk (B) and p-Akt (D) were positive in the tumorous epithelium. Bars: 50 μm.


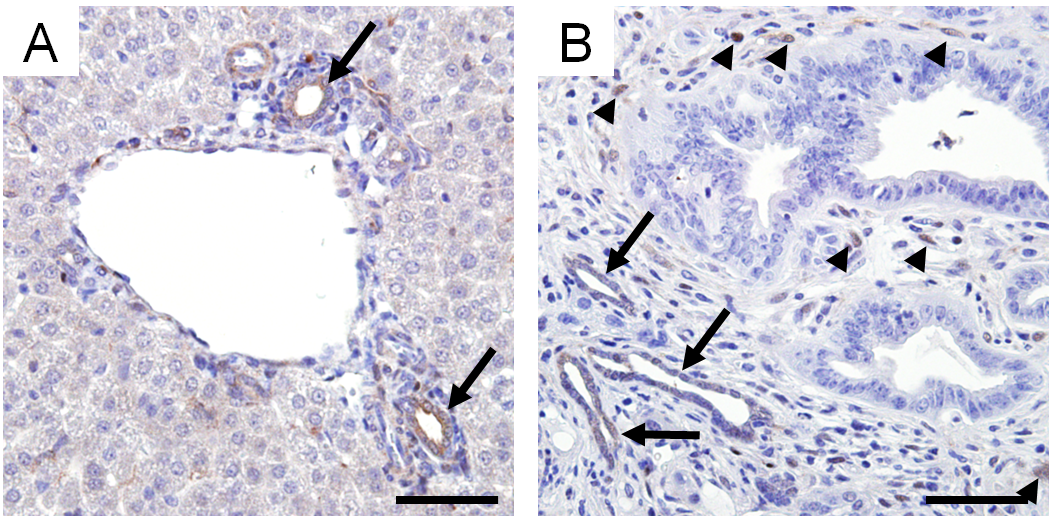


**Supplementary Figure S3.** Representative pictures of immunohistochemical staining of Pten in the liver of control mice (*Alb-Cre^+^*) (A) and *AKP* mice (B). (A) Pten was moderately expressed in the intrahepatic bile ducts (arrows) in control mice. (B) Pten expression was negative in the tumor cells in ICC lesions but positive in non-tumorous bile ducts (arrows) and some of the stromal cells (arrowheads) in *AKP* mice at 7 months of age. Bars: 50 μm.

**
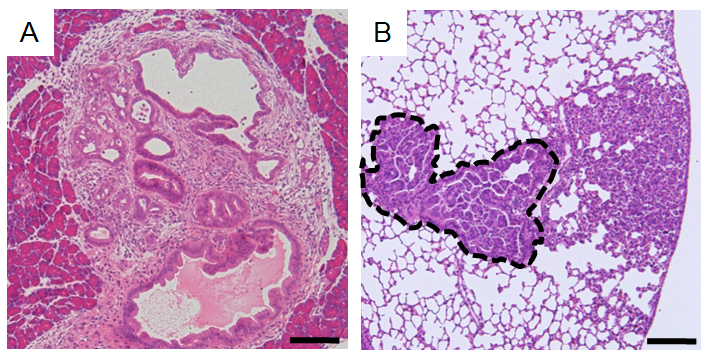
**

**Supplementary Figure S4.** Pancreas and lung lesions in the *K^ER^KPP* mice 4 weeks after TMX treatment. (A) Papillary hyperplastic change of pancreatic ducts and PanIN-like lesions were observed in the pancreata. (B) Papillary hyperplastic change of bronchial epithelia (dashed line) accompanying obstructive pneumonia was seen throughout the lung. Bar: 100 μm.

| **Supplementary Table S1. Primers used in this study** | |
| --- | --- |
| Primer | Sequence (5' to 3') |
| *Cre*-F | GCATTACCGGTCGATGCAACGAGTGATGAG |
| *Cre*-R | GAGTGAACGAACCTGGTCGAAATCAGTGCG |
| *LSL-Kras*-F | CCTTTACAAGCGCACGCAGACTGTAGA |
| *LSL-Kras*-R | AGGTAGCCACCATGGCTTGAGTAAGTCTGCA |
| *Ptenflox*-F | GTGAAAGTGCCCCAACATAAGG |
| *Ptenflox*-R | CTCCCACCAATGAACAAACAGTC |
| *mTmG*-F | CTCTGCTGCCTCCTGGCTTCT |
| *mTmG*-R1 | TCAATGGGCGGGGGTCGTT |
| *mTmG*-R2 | CGAGGCGGATCACAAGCAATA |
